# Comprehensive Study of Lipophilic Compounds from Various Cereal Straws (Wheat, Triticale, Rye, and Tritordeum)—a Promising Source of Valuable Phytochemicals

**DOI:** 10.1021/acs.jafc.4c12445

**Published:** 2025-03-17

**Authors:** Javier Benito, Gisela Marques, Francisco Barro, Ana Gutiérrez, José C. del Río, Jorge Rencoret

**Affiliations:** 1 Instituto de Recursos Naturales y Agrobiología de Sevilla (IRNAS-CSIC), Reina Mercedes 10, Seville E-41012, Spain; 2 Instituto de Agricultura Sostenible (IAS-CSIC), Córdoba E-14004, Spain

**Keywords:** lipids, GC−MS, agricultural residue, free sterols, diketones, waste valorization, fatty acids

## Abstract

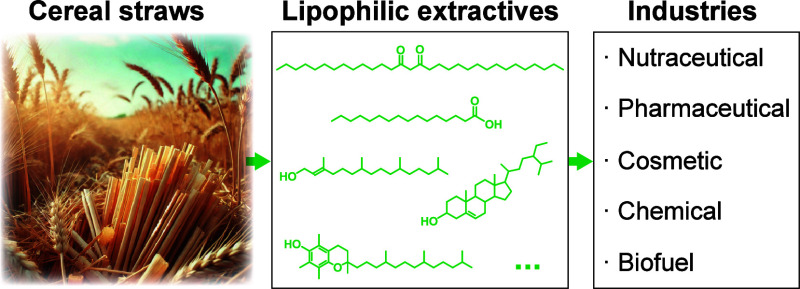

The lipid compositions of wheat, triticale, rye, and tritordeum straws were thoroughly analyzed using gas chromatography–mass spectrometry (GC–MS). The major lipophilic compounds identified included *n*-fatty acids (1185–3538 mg/kg), β-diketones (891–2043 mg/kg), steroid compounds (1358–1954 mg/kg), high molecular-weight esters (444–1560 mg/kg), and *n*-fatty alcohols (402–1825 mg/kg). Additionally, smaller amounts of *n*-alkanes (140–574 mg/kg), phytol and phytyl esters (106–358 mg/kg), 2-hydroxyfatty acids (77–155 mg/kg), acylglycerides (41–277 mg/kg), tocopherols and tocopheryl esters (21–67 mg/kg), and *n*-aldehydes (10–23 mg/kg) were detected. The abundance and wide diversity of lipophilic compounds present in these agricultural residues highlight their great potential as a rich source of valuable phytochemicals for various industrial applications, positioning cereal straws as highly attractive feedstocks in the context of the lignocellulosic biorefinery.

## 1 Introduction

From ancient civilizations to modern societies, cereals have served as dietary staples and are considered the most important crops in world agriculture. Their significance stems not only from their ability to provide more food energy than do any other crop, but also from their longstanding role as livestock feed since the beginning of civilization.^1^ Cereals owe their success to several key advantages: exceptional adaptability to different environments, high grain yields, and ease of harvesting and storage. In 2022, global cereal production amounted to 2942 million tons,^2^ considering only the main crops such as wheat, maize, rice, rye, oat, and barley. With the exponential growth of the world population, production is expected to continue rising.^3^

The harvesting of cereal crops generates large quantities of straws, which can account for up to half of the plant’s total weight. Cereal straws, traditionally considered agricultural waste, are now recognized for their potential in biorefineries due to their lignocellulosic composition. Typically, cereal straws consist of 34–38% cellulose, 27–32% hemicelluloses, 12–17% lignin, 5–7% ash, and 2–4% lipids.^4−6^ Their abundance, low cost, and widespread availability make them an exceptional feedstock for meeting the needs of other industries, including pharmaceuticals, cosmetics, biofuels, or the production of high-value chemicals, in the context of the lignocellulose biorefinery.^4,7−9^ Traditionally, biomass conversion industries have focused on the carbohydrate fraction for paper or biofuel production and the lignin fraction for the production of chemicals and polymers.^10^ However, to establish a sustainable and competitive global biobased circular economy employing lignocellulosic biomass as a feedstock, it is crucial to utilize all components of the biomass.^11,12^ In this context, the lipophilic fraction of cereal straws, comprising a wide variety of chemical compounds such as hydrocarbons, fatty alcohols, fatty acids, steroids, aldehydes, and tocopherols, is currently gaining attention for its potential applications across various industries, including pharmaceuticals, nutraceuticals, cosmetics, food, and chemicals.^5,9,13^ For example, plant sterols such as sitosterol or campesterol are used extensively in the pharmaceutical and nutraceutical industries due to their cholesterol-lowering properties,^14^ anti-inflammatory effects, and their prevention of various types of cancer.^15^ Another highly valuable lipid family for the pharmaceutical and nutraceutical industries is tocopherols, especially α-tocopherol (vitamin E), a powerful antioxidant necessary for the maintenance of cell membranes and serving as protection against oxidative stress.^16^

The aim of this study was to conduct a detailed analysis of the lipophilic profile of straws from four cereal species: wheat (*Triticum durum*), triticale (×*Triticosecale* Wittmack*)*, rye (*Secale cereale*), and tritordeum (×*Tritordeum* Ascherson et Graebner). Wheat is among the most cultivated cereals worldwide, with durum wheat standing out as one of the most extensively cultivated varieties,^17^ and its lipophilic composition has already been explored.^4^ In contrast, triticale, a hybrid cereal derived from wheat and rye,^18^ remains underexplored, and to date, only one study has explored the lipophilic profile of triticale straw using supercritical carbon dioxide and hexane extractions, but its primary focus was on optimizing extraction methods rather than providing a comprehensive analysis of its lipophilic profile.^19^ Similarly, research on rye straw is limited and incomplete, with only a few studies offering partial insights into its lipophilic composition.^20,21^ Finally, as for tritordeum straw, a hybrid cereal resulting from the crossing of wheat and wild barley, its lipophilic profile has not yet been reported in the literature. Therefore, this study aims to fill these gaps by performing a comprehensive and comparative study of the lipophilic components in wheat, triticale, rye, and tritordeum straws. The findings will provide valuable insights for optimizing the valorization of these agricultural residues, highlighting their potential as valuable sources of a wide range of phytochemicals with significant industrial applications.

## 2 Materials and Methods

### 2.1 Cereal Straws

Wheat, triticale, rye, and tritordeum plants were cultivated under field conditions in Córdoba (Spain), in 2022. Upon reaching optimal maturity, the plants were harvested and the straws were carefully collected. The straws were air-dried at room temperature until they reached a constant weight. Subsequently, they were ground in an IKA knife mill with a 1 mm sieve to facilitate lipid extraction. For each sample, 5 g of milled straw was precisely weighted and subjected to Soxhlet extraction with acetone for 8 h. The resulting extracts were then evaporated to dryness using a rotary evaporator until a constant weight was achieved, yielding 2.2% for wheat, 3.7% for triticale, 2.5% for rye, and 2.5% for tritordeum straws based on a dry material. The lipophilic extractives were obtained by redissolving the dried acetone extract in chloroform, yielding 1.5% for wheat, 2.5% for triticale, 1.7% for rye, and 1.9% for tritordeum. Each experiment was performed in triplicate to ensure accuracy.

### 2.2 GC–MS Analyses

For the GC–MS analysis, the chloroform–soluble fraction was analyzed both in their nonderivatized form and after derivatization with *N*,*O*-bis(trimethylsilyl)trifluoroacetamide (BSTFA). The analyses were performed in a Shimadzu QP 2010 Ultra GC–MS equipment (Kyoto, Japan) using the experimental conditions described elsewhere.^22^ Compound identification was conducted by comparing their mass spectra with those in the NIST library and, when possible, by comparison with authentic standards. To determine the response factor for the different lipid families and to quantify individual compounds, a mixture of authentic standards (all purchased from Sigma-Aldrich) was used in a concentration range between 0.3 and 1.1 mg/mL, including triacontane (98%), cholesta-3,5-diene (95%), palmitic acid (99%), 1-octacosanol (99%), 5α-cholestan-3-one (98%), sitosterol (99%), sitosteryl 3β-d-glucopyranoside (75%), cholesteryl linoleate (98%), *rac*-glycerol 1-myristate (99%), 1,3-dipalmitin (99%), and glyceryl tripalmitate (99%). Quantification results were given as the mean of three replicates to ensure accuracy and replicability.

## 3 Results and Discussion

### 3.1 Lipophilic Profile of the Cereal Straws

The acetone extraction yields from wheat, rye, and tritordeum straws were similar, ranging from 2.2 to 2.5% of the dry material, with triticale showing a significantly higher yield (3.7%). However, the lipophilic content, measured as the chloroform–soluble fraction, was lower, accounting for 1.5% in wheat, 2.5% in triticale, 1.7% in rye, and 1.9% in tritordeum. The chloroform–soluble fraction was subjected to GC–MS analysis, both underivatized and after derivatization with BSTFA to enhance the volatility of less volatile compounds. The use of medium-length high-temperature capillary GC columns, combined with the methodology described in previous studies,^23,24^ enabled the simultaneous identification of a wide range of lipids within a single chromatogram. These lipids ranged from low molecular-weight compounds, such as *n*-alkanes and *n*-fatty acids, to high molecular-weight compounds, including waxes and triglycerides.

**Figure 1:**
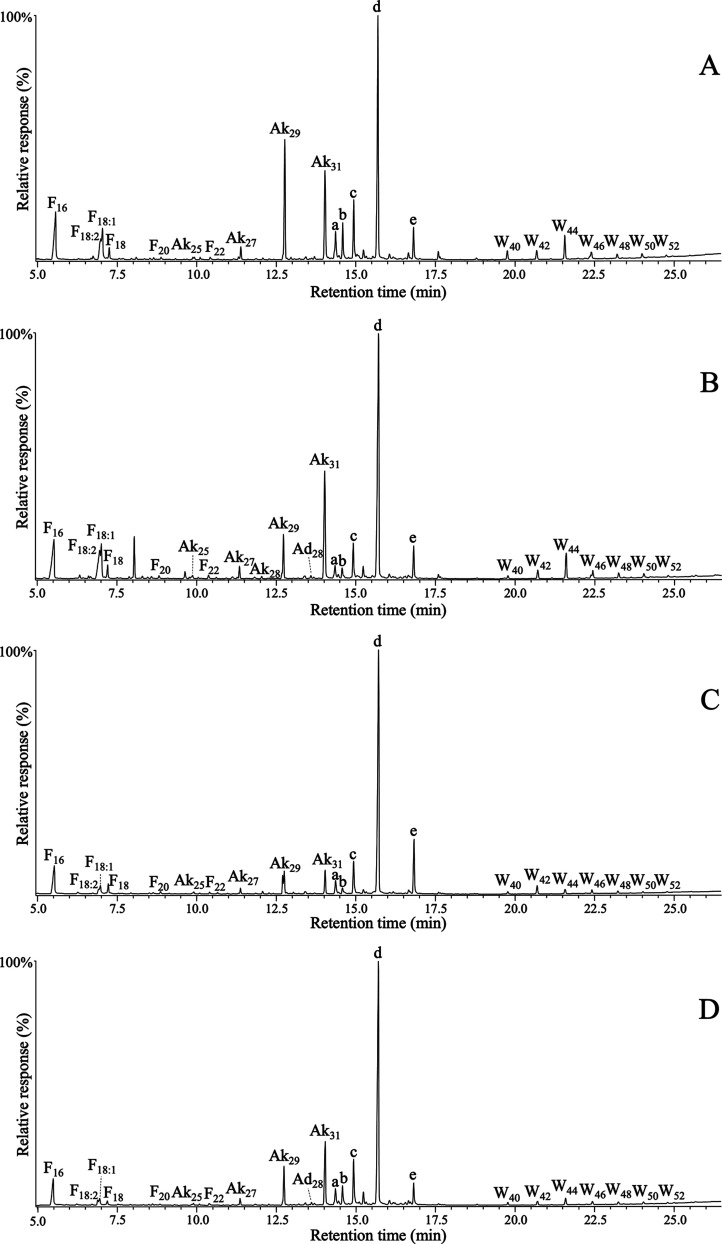
GC–MS chromatograms of the underivatized chloroform extracts from wheat (A), triticale (B), rye (C), and tritordeum (D) straws. Labels for selected compounds are F(n), *n*-fatty acids; Ak(n), *n*-alkanes; Ad(n), *n*-aldehydes; Ah(n), *n*-fatty alcohols; W(n), high molecular-weight esters; a, campesterol; b, stigmasterol; c, sitosterol; d, hentriacontane-14,16-dione; e, 25-hydroxyhentriacontane-14,16-dione.

**Figure 2:**
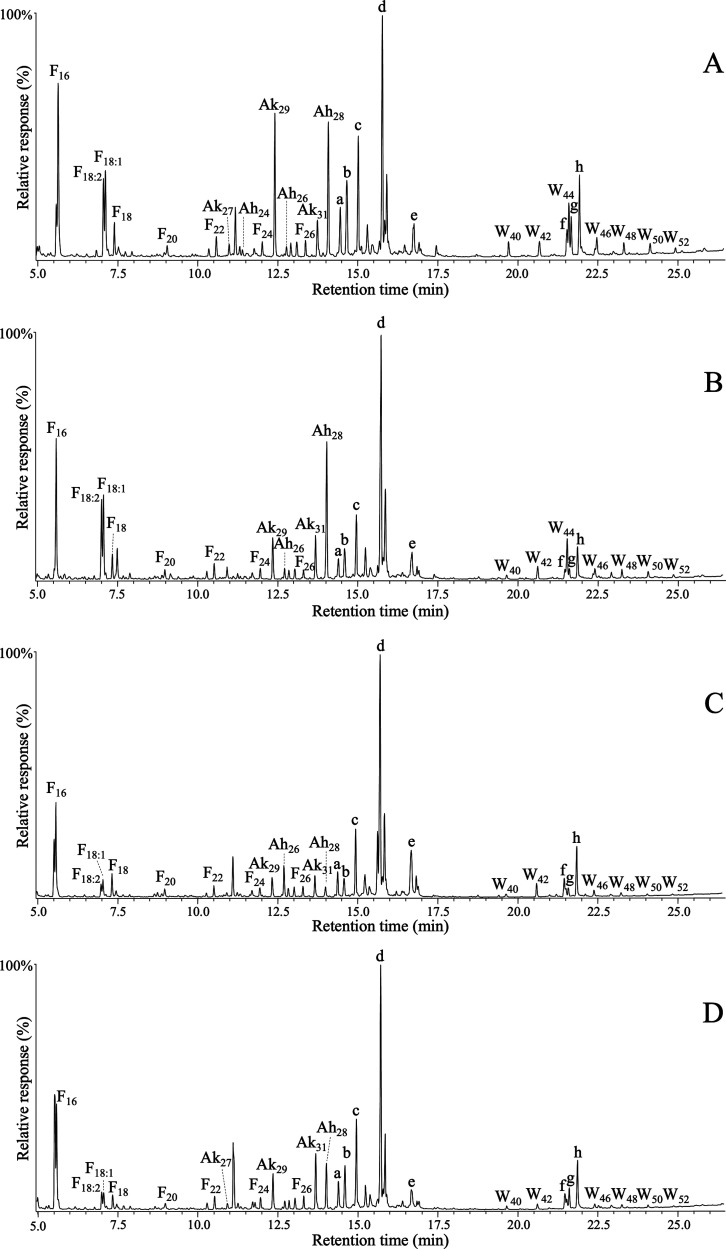
GC–MS chromatograms of the TMS-derivatized chloroform extracts from wheat (A), triticale (B), rye (C), and tritordeum (D) straws. Labels for selected compounds are F(n), *n*-fatty acids; Ak(n), *n*-alkanes; Ad(n), *n*-aldehydes; Ah(n), *n*-fatty alcohols; W(n), high molecular weight esters; a, campesterol; b, stigmasterol; c, sitosterol; d, hentriacontane-14,16-dione; e, 25-hydroxyhentriacontane-14,16-dione; f, campesteryl 3β-d-glucopyranoside; g, stigmasteryl 3β-d-glucopyranoside; h, sitosteryl 3β-d-glucopyranoside.

**Figure 3:**
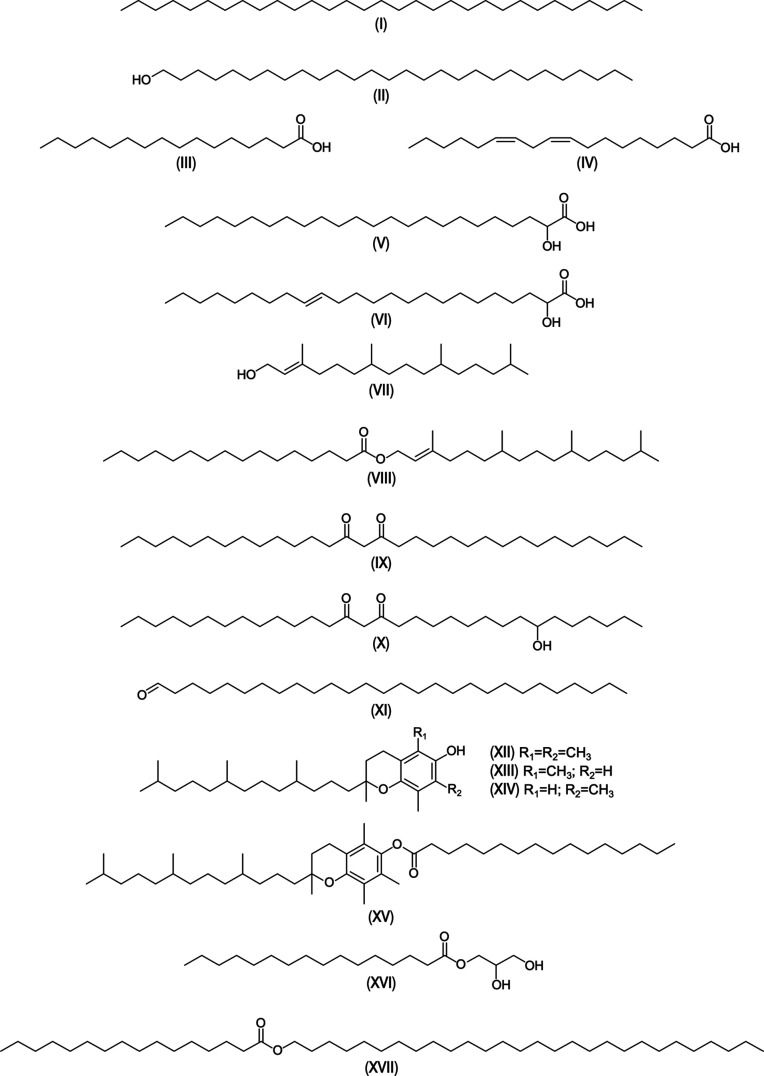
Chemical structures of representative aliphatic compounds from various lipid families identified in the cereal straws, as referred to in the text. **I**, *n*-hentriacontane; **II**, *n*-octacosanol; **III**, *n*-hexadecanoic acid; **IV**, *cis,cis*-9,12-octadecadienoic acid; **V**, 2-hydroxytetracosanoic acid; **VI**, 2-hydroxy-15-tetracosenoic acid; **VII**, phytol; **VIII**, phytyl hexadecanoate; **IX**, hentriacontane-14,16-dione; **X**, 25-hydroxyhentriacontane-14,16-dione; **XI**, *n*-octacosanal; **XII**, α-tocopherol; **XIII**, β-tocopherol; **XIV**, γ-tocopherol; **XV**, α-tocopheryl hexadecanoate; **XVI**, 1-monopalmitin; **XVII**, hexadecanoic acid, octacosyl ester.

Figures 1 and 2 show the chromatograms of the lipophilic extracts underivatized and after TMS-ether derivatization, respectively. A wide array of lipophilic compounds was identified and classified into two primary groups. The first group comprises aliphatic compounds, including *n*-alkanes; *n*-aldehydes; β-diketones; *n*-fatty acids; *n*-hydroxy fatty acids; mono-, di-, and triglycerides; *n*-fatty alcohols; phytols; tocopherols; and high-molecular-weight ester (waxes). The second group consists of steroid compounds, including free sterols, sterol hydrocarbons, sterol ketones, sterol glycosides, and sterol esters. The identity and abundance of these lipophilic compounds, expressed in milligrams per kilogram on a dry-weight basis, are detailed in Table 1. Representative structures of the most abundant compounds are shown in Figures 3 (for aliphatic compounds) and 4 (for steroid compounds). Figure 5 shows a comparison of the abundance of the main lipophilic compounds identified in the different cereal straws in terms of total abundance (mg/kg).

**Figure 4:**
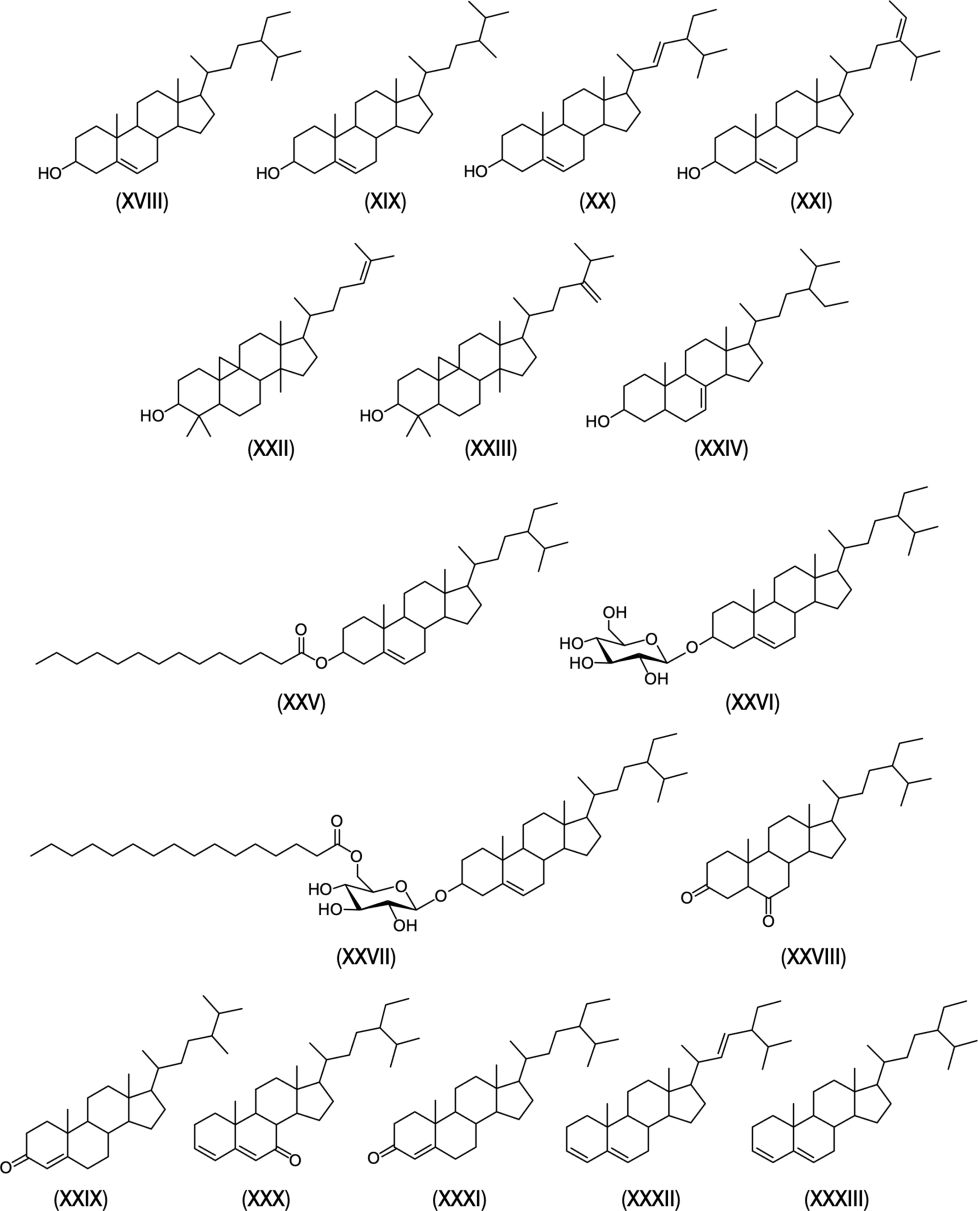
Chemical structures of representative steroid compounds from various lipid families identified in the cereal straws, as referred to in the text. **XVIII**, sitosterol; **XIX**, campesterol; **XX**, stigmasterol; **XXI**, Δ^5^-avenasterol; **XXII**, cycloartenol; **XXIII**, 24-methylenecycloartanol; **XXIV**, Δ^7^-stigmastenol; **XXV**, sitosteryl tetradecanoate; **XXVI**, sitosteryl 3β-d-glucopyranoside; **XXVII**, sitosteryl (6′-*O*-palmitoyl) 3β-d-glucopyranoside; **XXVIII**, stigmastane-3,6-dione; **XXIX**, ergost-4-en-3-one; **XXX**, stigmasta-3,5-dien-7-one; **XXXI**, stigmast-4-en-3-one; **XXXII**, stigmasta-3,5,22-triene; **XXXIII**, stigmasta-3,5-diene.

**Figure 5:**
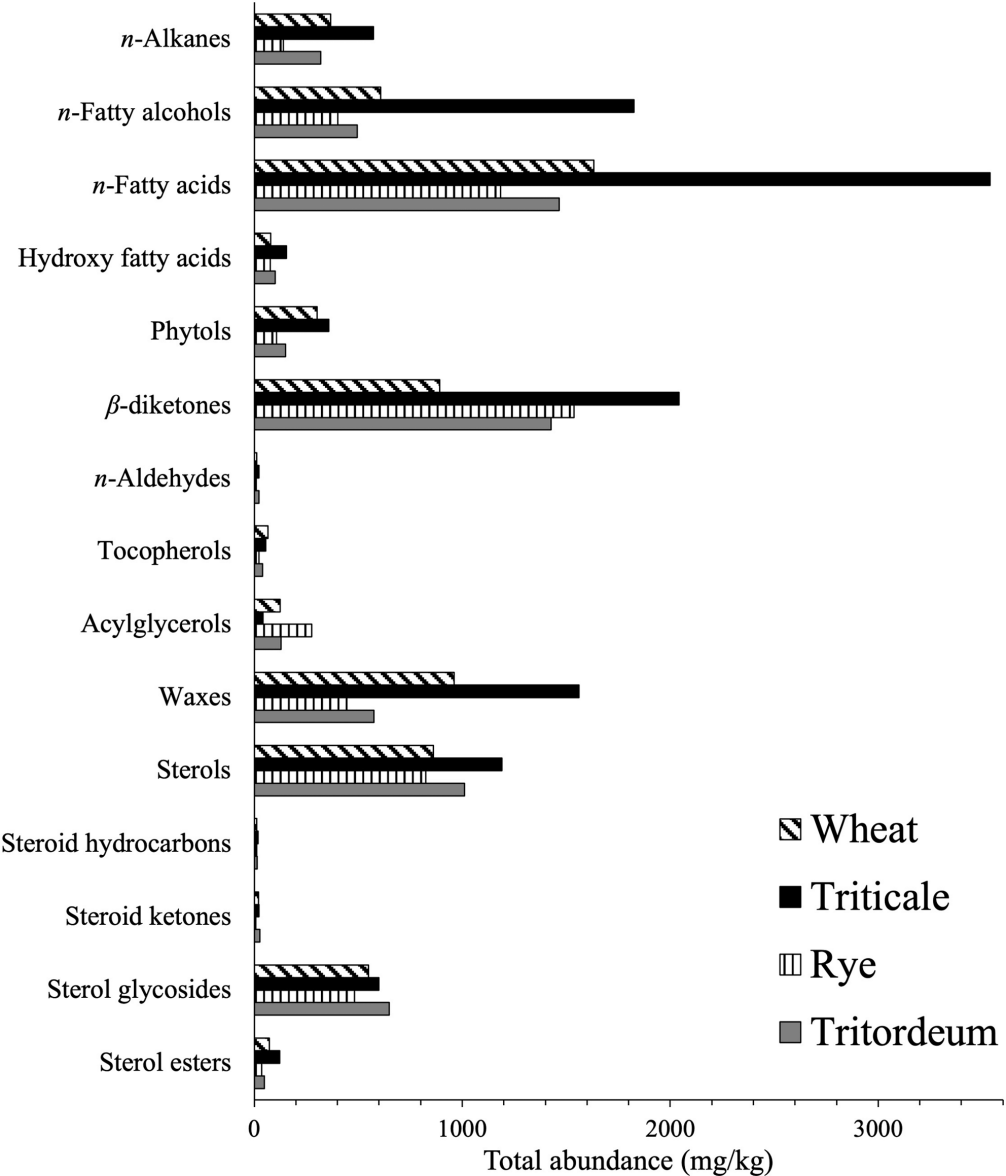
Total abundance (mg/kg, on a dry basis) of the main families of lipophilic compounds identified in the chloroform extracts of the cereal straws analyzed.

**Table 1:** Identities and Abundances (mg/kg) of the Lipophilic Compounds Identified in Wheat, Triticale, Rye, and Tritordeum Straws

**compounds**	**wheat**	**triticale**	**rye**	**tritordeum**
***n-*alkanes**	366 ± 5	574 ± 34	140 ± 3	319 ± 48
*n*-tricosane	1 ± 0	8 ± 0	tr.	1 ± 0
*n*-pentacosane	4 ± 0	13 ± 1	3 ± 0	5 ± 1
*n*-hexacosane	1 ± 0	5 ± 1	1 ± 0	1 ± 0
*n*-heptacosane	21 ± 1	50 ± 9	12 ± 0	17 ± 5
*n*-octacosane	3 ± 1	5 ± 0	3 ± 0	2 ± 0
*n*-nonacosane	193 ± 1	155 ± 10	53 ± 2	104 ± 10
*n*-triacontane	3 ± 0	5 ± 0	4 ± 0	2 ± 1
*n*-hentriacontane (**I**)	125 ± 1	292 ± 11	53 ± 1	152 ± 25
*n*-tritriacontane	13 ± 1	34 ± 2	10 ± 0	29 ± 4
*n*-pentatriacontane	2 ± 0	5 ± 0	1 ± 0	6 ± 2
***n*-fatty alcohols**	607 ± 13	1825 ± 71	402 ± 22	495 ± 41
*n*-eicosanol	6 ± 1	10 ± 1	1 ± 0	6 ± 1
*n*-docosanol	7 ± 0	32 ± 2	8 ± 0	42 ± 4
*n*-tetracosanol	16 ± 2	38 ± 3	27 ± 1	31 ± 2
*n*-hexacosanol	31 ± 0	101 ± 2	217 ± 10	48 ± 5
*n*-octacosanol (**II**)	478 ± 8	1485 ± 58	71 ± 8	291 ± 23
*n*-triacontanol	40 ± 1	94 ± 3	29 ± 2	35 ± 4
*n*-dotriacontanol	29 ± 1	57 ± 2	49 ± 1	42 ± 2
***n*-fatty acids**	1631 ± 66	3538 ± 81	1185 ± 63	1463 ± 86
*n*-tetradecanoic acid	82 ± 4	116 ± 5	108 ± 7	116 ± 6
*n*-hexadecanoic acid (**III**)	517 ± 28	810 ± 10	416 ± 21	475 ± 15
*n*-heptadecanoic acid	8 ± 1	30 ± 3	17 ± 0	12 ± 2
*cis*,*cis*-9,12-octadecadienoic acid (**IV**)	210 ± 1	653 ± 9	66 ± 4	89 ± 9
*cis*-9-octadecenoic acid	229 ± 10	645 ± 9	67 ± 4	79 ± 5
*n*-octadecanoic acid	91 ± 0	183 ± 6	110 ± 10	84 ± 3
*n*-nonadecanoic acid	18 ± 2	80 ± 2	22 ± 1	14 ± 1
*n*-eicosanoic acid	31 ± 3	140 ± 9	40 ± 0	41 ± 3
*n*-heneicosanoic acid	6 ± 0	13 ± 1	8 ± 1	16 ± 1
*n*-docosanoic acid	75 ± 4	138 ± 5	55 ± 3	62 ± 7
*n*-tricosanoic acid	19 ± 2	73 ± 4	19 ± 0	41 ± 3
*n*-tetracosanoic acid	60 ± 3	84 ± 1	42 ± 4	59 ± 6
*n*-pentacosanoic acid	7 ± 1	15 ± 0	11 ± 2	11 ± 2
*n*-hexacosanoic acid	38 ± 1	65 ± 3	45 ± 2	67 ± 1
*n*-heptacosanoic acid	9 ± 0	18 ± 1	5 ± 0	13 ± 2
*n*-octacosanoic acid	156 ± 1	218 ± 1	72 ± 0	146 ± 10
*n*-triacontanoic acid	60 ± 4	190 ± 10	33 ± 3	106 ± 8
*n*-dotriacontanoic acid	13 ± 1	49 ± 1	45 ± 1	25 ± 1
*n*-tetratriacontanoic acid	2 ± 0	10 ± 1	4 ± 0	7 ± 1
**2-hydroxy fatty acids**	80 ± 4	155 ± 6	77 ± 5	99 ± 11
2-hydroxydocosanoic acid	9 **±** 0	23 **±** 1	15 ± 0	19 ± 2
2-hydroxytricosanoic acid	23 ± 2	38 ± 0	16 ± 1	18 ± 4
2-hydroxytetracosanoic acid (**V**)	23 **±** 0	43 **±** 0	22 ± 2	31 ± 3
2-hydroxy-15-tetracosenoic acid (**VI**)	19 **±** 1	38 **±** 4	20 ± 1	22 ± 1
2-hydroxyhexacosanoic acid	6 **±** 1	13 **±** 1	6 ± 0	9 ± 1
**phytol and phytyl esters**	302 ± 21	358 ± 18	106 ± 10	151 ± 7
phytol (**VII**)	57 ± 1	89 ± 1	26 ± 1	38 ± 2
phytyl tetradecanoate	26 ± 0	72 ± 2	11 ± 1	18 ± 1
phytyl hexadecanoate (**VIII**)	83 ± 8	73 ± 4	41 ± 4	53 ± 3
phytyl linoleate	101 ± 10	78 ± 4	15 ± 2	23 ± 0
phytyl oleate	24 ± 1	31 ± 4	7 ± 1	7 ± 1
phytyl octadecanoate	7 ± 1	7 ± 2	2 ± 0	8 ± 0
phytyl eicosanoate	4 ± 0	6 ± 1	4 ± 1	4 ± 0
**β-diketones**	891 ± 19	2043 ± 103	1538 ± 70	1427 ± 123
hentriacontane-14,16-dione (**XI**)	798 ± 9	1879 ± 83	1308 ± 48	1333 ± 95
25-hydroxy-hentriacontane-14,16-dione (**X**)	93 ± 10	163 ± 20	230 ± 22	94 ± 28
*n***-aldehydes**	12 ± 1	23 ± 4	10 ± 1	22 ± 2
*n*-docosanal	2 ± 0	2 ± 0	1 ± 0	3 ± 0
*n*-tetracosanal	2 ± 0	2 ± 0	1 ± 0	2 ± 0
*n*-hexacosanal	3 ± 0	4 ± 1	5 ± 1	5 ± 1
*n*-octacosanal (**XI**)	4 ± 1	11 ± 3	1 ± 0	9 ± 1
*n-*triacontanal	1 ± 0	4 ± 1	2 ± 0	3 ± 0
**tocopherols and tocopheryl esters**	67 ± 4	55 ± 7	21 ± 4	40 ± 3
α-tocopherol (**XII**)	39 ± 3	38 ± 1	8 ± 2	22 ± 1
β-tocopherol (**XIII**)	5 ± 1	3 ± 1	2 ± 0	3 ± 0
γ-tocopherol (**XIV**)	8 ± 0	7 ± 0	5 ± 1	6 ± 1
α-tocopheryl dodecanoate	tr.	tr.	1 ± 0	2 ± 0
α-tocopheryl tetradecanoate	2 ± 0	1 ± 0	tr.	1 ± 0
α-tocopheryl hexadecanoate (**XV**)	5 ± 0	2 ± 0	3 ± 1	1 ± 0
β-tocopheryl dodecanoate	4 ± 0	2 ± 0	1 ± 0	3 ± 0
β-tocopheryl tetradecanoate	4 ± 0	2 ± 0	tr.	1 ± 0
β-tocopheryl hexadecanoate	tr.	tr.	1 ± 0	1 ± 1
**monoglycerides**^a^	26 ± 1	75 ± 2	23 ± 1	40 ± 2
1-monopalmitin (1-P) **(XVI)**	11 ± 1	36 ± 1	12 ± 1	18 ± 0
1-monolinolein (1-L)	4 ± 0	13 ± 1	3 ± 0	5 ± 1
1-monoolein (1-O)	3 ± 0	5 ± 0	2 ± 0	2 ± 0
2,3-dihydroxypropyl octadecanoate	2 ± 0	5 ± 0	1 ± 0	7 ± 1
2,3-dihydroxypropyl eicosanoate	tr.	tr.	tr.	tr.
2,3-dihydroxypropyl docosacanoate	1 ± 0	4 ± 0	1 ± 0	2 ± 0
2,3-dihydroxypropyl tetracosanoate	2 ± 0	4 ± 0	2 ± 0	2 ± 0
2,3-dihydroxypropyl hexacosanoate	tr.	tr.	tr.	tr.
2,3-dihydroxypropyl octacosanoate	2 ± 0	3 ± 0	1 ± 0	3 ± 0
2,3-dihydroxypropyl triacontanoate	1 ± 0	3 ± 0	1 ± 0	1 ± 0
**diglycerides**^a^	28 ± 1	60 ± 6	18 ± 1	30 ± 4
1,2-Dg35 (1,2-P2)	1 ± 0	3 ± 0	5 ± 0	4 ± 0
1,3-Dg35 (1,3-P2)	1 ± 0	4 ± 1	3 ± 0	6 ± 1
1,2-Dg37 (1,2-PO + 1,2-PL)	2 ± 0	8 ± 1	1 ± 0	4 ± 0
1,3-Dg37 (1,3-PO + 1,3-PL)	7 ± 0	20 ± 2	5 ± 1	4 ± 1
1,2-Dg39 (1,2-O2 + 1,2-L2 + 1,2-OL)	12 ± 1	10 ± 1	3 ± 0	6 ± 1
1,3-Dg39 (1,3-O2 + 1,3-L2 + 1,3-OL)	5 ± 0	15 ± 1	1 ± 0	6 ± 1
**triglycerides**^a^	72 ± 3	142 ± 5	n.d.	60 ± 4
Tg53 (P2O + P2S + P2L)	18 ± 1	29 ± 2	n.d.	14 ± 0
Tg55 (PL2 + PLS + PO2 + PS2 + PLO + POS)	48 ± 1	83 ± 1	n.d.	39 ± 4
Tg57 (L3 + O3)	6 ± 1	30 ± 2	n.d.	7 ± 0
**high-molecular-weight esters**	959 ± 42	1560 ± 97	444 ± 30	572 ± 34
esters C_38_	21 ± 3	23 ± 1	5 ± 0	17 ± 1
esters C_39_	5 ± 1	3 ± 0	1 ± 0	3 ± 0
esters C_40_	128 ± 4	89 ± 5	48 ± 2	50 ± 3
esters C_41_	11 ± 1	9 ± 1	4 ± 1	7 ± 1
esters C_42_	120 ± 4	218 ± 14	149 ± 9	94 ± 2
esters C_43_	19 ± 1	32 ± 4	5 ± 1	9 ± 1
esters C_44_	311 ± 3	699 ± 32	81 ± 6	158 ± 15
esters C_45_	15 ± 1	16 ± 0	5 ± 1	13 ± 0
esters C_46_	108 ± 10	104 ± 3	64 ± 6	65 ± 4
esters C_48_	75 ± 4	142 ± 13	42 ± 2	58 ± 1
esters C_50_	71 ± 5	117 ± 11	24 ± 0	49 ± 2
esters C_52_	35 ± 3	50 ± 6	16 ± 2	26 ± 1
esters C_54_	16 ± 1	26 ± 1	n.d.	13 ± 0
esters C_56_	24 ± 1	32 ± 6	n.d.	10 ± 3
**free sterols**	860 ± 12	1192 ± 26	824 ± 82	1010 ± 82
cholesterol	20 ± 0	17 ± 1	8 ± 2	24 ± 3
sitosterol (**XVIII**)	417 ± 0	684 ± 9	496 ± 48	563 ± 49
campesterol (**XIX**)	196 ± 1	224 ± 6	202 ± 12	189 ± 9
stigmasterol (**XX**)	161 ± 5	105 ± 5	48 ± 2	147 ± 10
stigmastanol	17 ± 2	73 ± 3	39 ± 7	50 ± 4
Δ^5^-avenasterol (**XXI**)	12 ± 2	42 ± 1	n.d.	11 ± 2
cycloartenol (**XXII**)	18 ± 2	12 ± 1	tr.	5 ± 0
24-methylenecycloartanol (**XXIII**)	2 ± 0	1 ± 0	tr.	3 ± 0
Δ^7^-stigmastenol (**XXIV**)	n.d.	n.d.	10 ± 1	n.d.
β-amyrin	17 ± 1	30 ± 2	21 ± 5	18 ± 5
**sterol esters**	73 ± 4	122 ± 11	35 ± 2	47 ± 1
campesteryl tetradecanoate	tr.	4 ± 1	3 ± 0	3 ± 0
campesteryl hexadecanoate	6 ± 0	11 ± 1	1 ± 0	2 ± 0
campesteryl oleate + campesteryl linoleate	6 ± 1	9 ± 1	tr.	1 ± 0
stigmasteryl tetradecanoate	5 ± 1	5 ± 0	1 ± 0	3 ± 0
stigmasteryl hexadecanoate	6 ± 1	16 ± 2	tr.	3 ± 0
stigmasteryl oleate + stigmasteryl linoleate	tr.	tr.	tr.	tr.
sitosteryl tetradecanoate (**XXV**)	33 ± 0	32 ± 1	18 ± 1	25 ± 0
sitosteryl hexadecanoate	10 ± 0	22 ± 2	5 ± 0	6 ± 1
sitosteryl oleate + sitosteryl linoleate	7 ± 1	21 ± 3	7 ± 1	4 ± 0
**sterol glycosides**	550 ± 24	599 ± 15	481 ± 18	649 ± 12
cholesteryl 3β-d-glucopyranoside	8 ± 1	10 ± 1	3 ± 0	11 ± 2
campesteryl 3β-d-glucopyranoside	94 ± 10	108 ± 0	107 ± 10	129 ± 2
stigmasteryl 3β-d-glucopyranoside	115 ± 3	89 ± 3	58 ± 3	114 ± 1
sitosteryl 3β-d-glucopyranoside (**XXVI**)	273 ± 6	328 ± 9	279 ± 0	335 ± 4
Δ^5^-avenasteryl 3β-d-glucopyranoside	15 ± 1	7 ± 0	n.d.	9 ± 1
Δ^7^-stigmastenyl 3β-d-glucopyranoside	n.d.	n.d.	10 ± 4	n.d.
campesteryl (6′-*O*-palmitoyl)-3β-d-glucopyranoside	10 ± 1	12 ± 1	6 ± 1	11 ± 0
stigmasteryl (6′-*O*-palmitoyl)-3β-d-glucopyranoside	11 ± 1	13 ± 1	3 ± 0	9 ± 1
sitosteryl (6′-*O*-palmitoyl)-3β-d-glucopyranoside (**XXVII**)	24 ± 1	32 ± 0	15 ± 0	31 ± 1
**steroid ketones**	20 ± 1	22 ± 2	7 ± 2	26 ± 2
stigmastane-3,6-dione (**XXVIII**)	4 ± 0	9 ± 1	3 ± 1	22 ± 1
ergost-4-en-3-one (**XXIX**)	10 **±** 1	8 ± 1	3 ± 1	11 ± 1
stigmasta-3,5-dien-7-one (**XXX**)	3 ± 0	1 ± 0	tr	2 ± 0
stigmast-4-en-3-one (**XXXI**)	3 ± 0	3 ± 0	1 ± 0	2 ± 0
**steroid hydrocarbons**	12 ± 1	19 ± 4	11 ± 2	14 ± 2
stigmasta-3,5,22-triene (**XXXII**)	6 ± 1	11 ± 2	7 ± 1	7 ± 1
stigmasta-3,5-diene (**XXXIII**)	6 ± 0	8 ± 2	4 ± 1	7 ± 1

a Labels for mono-, di-, and triglycerides: P, palmitic acid; L, linoleic acid; O, oleic acid; S, stearic acid.

In wheat straw, *n*-fatty acids were the most abundant lipophilic family (1631 mg/kg; 24.9% of all of the identified compounds), followed by high molecular-weight esters (959 mg/kg; 14.7%), β-diketones (891 mg/kg; 13.6%), and free sterols (860 mg/kg; 13.1%). Triticale straw was characterized by presenting the highest contents of *n*-fatty acids (3538 mg/kg; 28.6%), β-diketones (2043 mg/kg; 16.5%), *n*-fatty alcohols (1825 mg/kg; 14.8%), and high molecular-weight esters (1560 mg/kg; 12.6%), which makes sense considering its higher total lipophilic content. Rye straw was particularly rich in β-diketones (1538 mg/kg; 28.9%), with significant amounts of *n*-fatty acids (1185 mg/kg; 22.2%), free sterols (824 mg/kg; 15.5%), and sterol glycosides (481 mg/kg; 9.0%). Lastly, the lipophilic profile of tritordeum straw was dominated by *n*-fatty acids (1463 mg/kg; 22.6%), followed by β-diketones (1427 mg/kg; 22.1%), free sterols (1010 mg/kg; 15.6%), and sterol glycosides (649 mg/kg; 10.0%), as depicted in the histograms of Figure 5.

### 3.2 Aliphatic Compounds

The aliphatic compounds identified in the straw samples included a variety of *n*-alkanes, *n*-aldehydes, β-diketones, *n*-fatty acids, *n*-hydroxy fatty acids, glycerides, *n*-fatty alcohols, phytols, tocopherols, and high molecular-weight esters. These compounds were found at different concentrations, with the highest levels found in triticale (10408 mg/kg), followed by tritordeum (4718 mg/kg), wheat (5041 mg/kg), and rye (3964 mg/kg) (Table 1).

*n*-Alkanes were present in considerable amounts in triticale (574 mg/kg; 4.6% of the total lipidic compounds), wheat (366 mg/kg; 5.6%), and tritordeum (319 mg/kg; 4.9%), whereas rye contained significantly lower amounts (140 mg/kg; 2.6%) (Figure 5). These compounds ranged from *n*-tricosane (C_23_) to *n*-pentatriacontane (C_35_), with *n*-nonacosane (C_29_) being the most abundant in wheat (193 mg/kg) and *n*-hentriacontane (C_31_; **I**) being predominant in triticale (292 mg/kg) and tritordeum (152 mg/kg); in rye, both alkanes were present in equal amounts (53 mg/kg), as shown in Table 1. These results are consistent with a previous study on wheat straw.^4^ In a previous study on triticale, only *n*-heptacosane (C_27_), *n*-nonacosane (C_29_), and *n*-hentriacontane (C_31_) were reported.^19^

*n*-Fatty alcohols were also present in notable amounts, with triticale showing the highest abundance (1825 mg/kg; 14.8% of the total identified lipophilic compounds). In wheat, tritordeum, and rye, fatty alcohols accounted for 607 mg/kg (9.3%), 495 mg/kg (7.6%), and 402 mg/kg (7.5%) (Figure 5). The *n*-fatty alcohols ranged from *n*-eicosanol (C_20_) to *n*-dotriacontanol (C_32_), with an even carbon atom number predominance. *n*-Octacosanol (C_28_; **II**) was the most abundant *n*-fatty alcohol, accounting for up to 1485 mg/kg in triticale, 478 mg/kg in wheat, and 291 mg/kg in tritordeum. However, in rye straw, the most abundant *n*-fatty alcohol was *n*-hexacosanol (C_26_), accounting for 217 mg/kg (Table 1). These findings are consistent with previous studies on wheat straw.^4^ In the case of triticale, only *n*-hexacosanol and *n*-octacosanol have been previously documented,^19^ while there is a lack of previous research reporting fatty alcohols in tritordeum and rye.

*n*-Fatty acids emerged as one of the most abundant lipid families in straw, contributing significantly to the total lipophilic content. They accounted for 3538 mg/kg (28.6% of all lipophilic compounds) in triticale, 1631 mg/kg (24.9%) in wheat, 1463 mg/kg (22.6%) in tritordeum, and 1185 mg/kg (22.2%) in rye, as depicted in Figure 5. The distribution of *n*-fatty acids ranged from *n*-tetradecanoic acid (C_14_) to *n*-tetratriacontanoic acid (C_34_), with a predominance of *n*-hexadecanoic acid (C_16_, palmitic acid, **III**), accounting for 810 mg/kg in triticale, 517 mg/kg in wheat, 475 mg/kg in tritordeum, and 416 mg/kg in rye. Moreover, significant amounts of unsaturated fatty acids, such as *cis,cis*-9,12-octadecadienoic acid (C_18:2_, linoleic acid, **IV**) and *cis*-9-octadecenoic acid (C_18:1_, oleic acid), were also identified, with triticale showing the highest levels (653 and 645 mg/kg, respectively), followed by wheat (210 and 229 mg/kg), tritordeum (89 and 79 mg/kg), and rye (66 and 67 mg/kg) (Table 1). A similar trend was observed in previous work for wheat.^4^ In contrast, previous studies have reported higher amounts of unsaturated fatty acids in triticale and rye, likely due to the different extraction methods.^19,20^

Minor amounts of 2-hydroxy fatty acids were also detected, ranging from 155 mg/kg (1.2% of all lipophilic compounds) in triticale to 99 mg/kg (1.5%) in tritordeum, 80 mg/kg (1.2%) in wheat, and 77 mg/kg (1.5%) in rye. The most abundant was 2-hydroxytetracosanoic acid (C_24_, **V**), found at 43 mg/kg in triticale, 31 mg/kg in tritordeum, 23 mg/kg in wheat, and 22 mg/kg in rye (Table 1). 2-Hydroxy fatty acids have been widely found in a variety of plants.^25,26^ Interestingly, an unsaturated 2-hydroxy fatty acid, namely, 2-hydroxy-15-tetracosenoic acid (**VI**), was detected for the first time in these straws, which accounted for 38 mg/kg in triticale straw, 22 mg/kg in tritordeum, 20 mg/kg in rye, and 19 mg/kg in wheat. This compound was previously reported in the Caribbean urchin *Tripneustes esculentus*.^27^

Phytol, an unsaturated diterpene alcohol, and its esters were also detected, accounting for 358 mg/kg (2.9% of the total lipophilic content) in triticale, 302 mg/kg (4.6%) in wheat, 151 mg/kg (2.3%) in tritordeum, and 106 mg/kg (2.0%) in rye (Figure 5). This is the first time that these compounds have been reported in cereal straws. Although the content of phytol (**VII**) was relatively low—89 mg/kg in triticale, 57 mg/kg in wheat, 38 mg/kg in tritordeum, and 26 mg/kg in rye—phytyl esters, formed by the esterification of phytol with different fatty acids, were more abundant. These esters ranged from phytyl tetradecanoate (C_14_) to phytyl eicosanoate (C_20_), including two unsaturated phytol esters, phytyl oleate (C_18:1_) and phytyl linoleate (C_18:2_). Phytyl hexadecanoate (C_16_; **VIII**) was the most abundant phytyl ester in tritordeum and rye, with concentrations of 53 and 41 mg/kg, respectively. In contrast, phytyl linoleate (C_18:2_) was the predominant one in wheat and triticale, with concentration of 101 and 78 mg/kg, respectively (Table 1). Phytol and its derivatives offer numerous health benefits, including antioxidant, anti-inflammatory, anticancer, and antimicrobial properties, and are used in the synthesis of vitamins E and K1,^28^ making them highly valuable in the pharmaceutical industry.^29,30^

**Figure 6:**
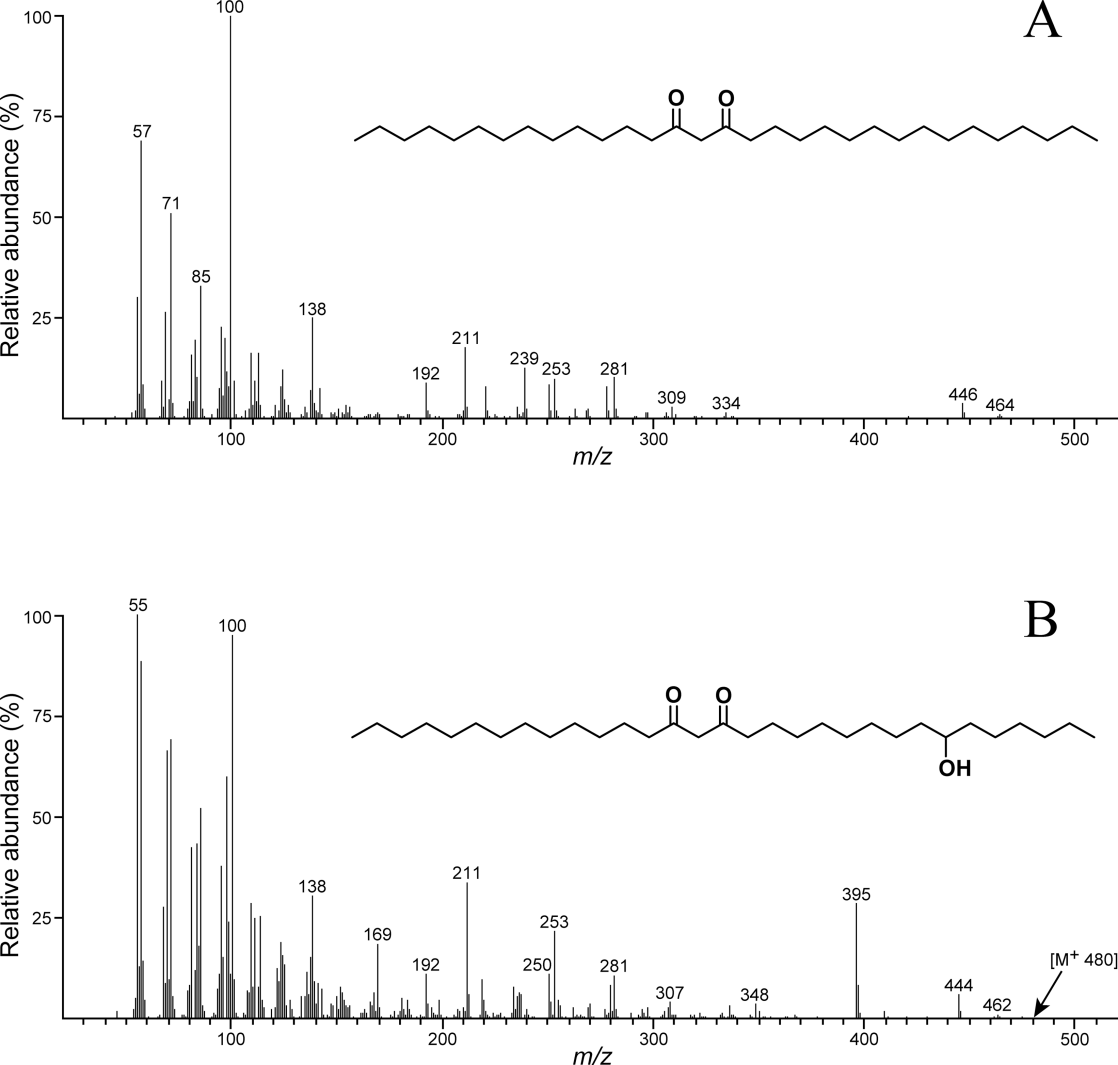
Mass spectra of hentriacontane-14,16-dione (A) and 25-hydroxyhentriacontane-14,16-dione (B), identified among the lipophilic extractives of the cereal straws.

β-Diketones constituted a significant portion of the lipophilic extractives in the cereal straws analyzed. They accounted for 2043 mg/kg (16.5% of the total lipophilic compounds) in triticale straw, 1538 mg/kg (28.9%) in rye, 1427 mg/kg (22.6%) in tritordeum, and 891 mg/kg (13.6%) in wheat. Two β-diketones were identified, hentriacontane-14,16-dione (**IX**) and 25-hydroxyhentriacontane-14,16-dione (**X**), with their mass spectra shown in Figure 6. The fragmentation pattern of hentriacontane-14,16-dione differed slightly from previous reports,^4^ likely due to the use of a different mass spectrometer detector (Ion-trap versus quadrupole). The molecular ion at *m*/*z* 464 suggests that the compound is a hentriacontanedione, while the fragments at *m*/*z* 211, 225, 239, and 251 correspond to cleavages at adjacent positions of the carbonyl groups at C14 and C16. This compound was found in relatively high amounts, with concentrations of 1879 mg/kg in triticale, 1333 mg/kg in tritordeum, 1308 mg/kg in rye, and 798 mg/kg in wheat (Table 1). Hentriacontane-14,16-dione has been previously reported in wheat straw.^4,31,32^ In contrast, the mass spectrum of 25-hydroxyhentriacontane-14,16-dione (Figure 6) lacks the molecular ion (*m*/*z* 480), likely due to the loss of a water molecule, resulting in a fragment at *m*/*z* 462. As with hentriacontane-14,16-dione, the fragments at *m*/*z* 211 and 253 are characteristic of the carbonyl groups at C14 and C16 positions, while the fragment at *m*/*z* 395 arises from a cleavage adjacent to the hydroxyl group at C-25. 25-Hydroxyhentriacontane-14,16-dione was present in lower amounts, with concentrations of 230 mg/kg in rye, 163 mg/kg in triticale, 94 mg/kg in tritordeum, and 93 mg/kg in wheat (Table 1). This compound has been previously detected in other grasses such as *L. arenarius* and some *Agropyron* species.^33,34^

β-Diketones are natural antioxidants with a broad spectrum of biological activities. A notable example is curcumin, a well-known diketone extensively studied for its health benefits, including its role in preventing cardiovascular and liver diseases, hypertension, and obesity.^35,36^ The characteristic keto–enol tautomerism inherent to β-diketones renders them highly versatile for applications in various industries. They serve as substrates for catalyst manufacturing, medicines, cosmetic and fuel additives, and even as chelating agents for environmental protection.^37^ The substantial presence of β-diketones in the lipid fraction of these cereal straws highlights the potential of these inexpensive and abundant agricultural residues as a renewable source for extracting this valuable family of lipophilic compounds. In previous studies on wheat straw,^4^ hentriacontane-14,16-dione was the second most abundant compound after *n*-octacosanol. In triticale, hentriacontane-14,16-dione was the most abundant compound detected, and 25-hydroxyhentriacontane-14,16-dione had only been found in the leaves of the plant.^38^

*n*-Aldehydes were also found in the cereal straws, albeit in minor amounts, accounting for 23 mg/kg (0.2% of the total lipophilic compounds) in triticale, 22 mg/kg (0.3%) in tritordeum, 12 mg/kg (0.2%) in wheat, and 10 mg/kg (0.2%) in rye, as shown in Figure 5. The *n*-aldehydes identified ranged from *n*-docosanal (C_22_) to *n*-triacontanal (C_30_), with *n*-octacosanal (C_28_; **XI**) being the most abundant in triticale (11 mg/kg), tritordeum (9 mg/kg), and wheat (4 mg/kg) straws. In contrast, the maximum for rye was *n*-hexacosanal, accounting for 5 mg/kg (Table 1). Although aldehydes have traditionally been overlooked due to their high reactivity and associated toxicity, recent studies have demonstrated their potential in the development of highly selective drugs^39^ and their excellent antibacterial properties.^40,41^

The analysis also revealed small amounts of both free and esterified tocopherols. Notably, this study is the first to confirm the presence of tocopherols in specific straw samples. Tocopherols accounted for 67 mg/kg (1.0% of the total lipophilic extract) in wheat, 55 mg/kg (0.4%) in triticale, 40 mg/kg (0.6%) in tritordeum, and 21 mg/kg (0.4%) in rye (Figure 5). Their identification was confirmed by comparing their mass spectra with previously published data.^42^ Among the tocopherols, α-tocopherol (**XII**) was the most abundant, with concentrations of 39 mg/kg in wheat, 38 mg/kg in triticale, 22 mg/kg in tritordeum, and 8 mg/kg in rye (Table 1). Additionally, β-tocopherol (**XIII**) and γ-tocopherol (**XIV**) were also detected, albeit in lower amounts. While α-, β-, and γ-tocopherols were found in free form, only α- and β-tocopherols were detected in esterified form, bound to various *n*-fatty acids. The esterified tocopherols ranged from α- and β-tocopheryl dodecanoate (C_12_) to α- and β-tocopheryl hexadecanoate (C_16_), with only even-numbered carbon chain homologues detected. Among these, α-tocopheryl hexadecanoate (**XV**) was the most abundant, especially in wheat (5 mg/kg) and rye (3 mg/kg) (Table 1). Tocopherols are widely known for their antioxidant properties and associated health benefits, as documented in numerous studies.^43,44^

Acylglycerols, including mono-, di-, and triglycerides, were detected in low amounts in the straw samples, accounting for 277 mg/kg (2.2% of the total lipophilic compounds) in triticale, 130 mg/kg (2.0%) in tritordeum, 126 mg/kg (1.9%) in wheat, and 41 mg/kg (0.8%) in rye (Figure 5). Monoglycerides accounted for 75 mg/kg in triticale, 40 mg/kg in tritordeum, 26 mg/kg in wheat, and 23 mg/kg in rye (Table 1). Among these, 1-monopalmitin was the most abundant, with a concentration of 36 mg/kg in triticale, 18 mg/kg in tritordeum, 12 mg/kg in rye, and 11 mg/kg in wheat (Table 1). On the other hand, diglycerides accounted for 60 mg/kg in triticale, 30 mg/kg in tritordeum, 28 mg/kg in wheat, and 18 mg/kg in rye (Table 1). These were identified as a mixture of compounds formed from different fatty acids (palmitic, oleic, and linoleic acids) attached to different positions on the glycerol backbone, with 1,3-isomers being more abundant than 1,2-isomers (Table 1). Lastly, triglycerides were the most abundant acylglycerols found among the straws, accounting for 142 mg/kg in triticale, 72 mg/kg in wheat, and 60 mg/kg in tritordeum, but they were not detected in rye straw. Triglycerides, as in the case of diglycerides, were composed of mixtures of palmitic, stearic, oleic, and linoleic acids. Three main peaks were identified, Tg53, Tg55, and Tg57. Peak Tg55, which included palmitoyldilinolein (PL2), palmitoyllinoleoylstearin (PLS) + palmitoyldiolein (PO2), palmitoyldistearin (PS2), palmitoyllinoleoylolein (PLO) + palmitoyloleoylstearin (POS), was the most abundant, accounting for 83 mg/kg in triticale, 48 mg/kg in wheat, and 39 mg/kg in tritordeum (Table 1).

High molecular-weight esters, commonly known as waxes, were among the most abundant aliphatic compounds identified in the four cereal straws. These waxes accounted for 1560 mg/kg (12.6% of the total lipophilic compounds) in triticale, 959 mg/kg (14.7%) in wheat, 572 mg/kg (8.9%) in tritordeum, and 444 mg/kg (8.4%) in rye (Figure 5). These compounds arise from the esterification of various *n*-fatty acids and *n*-fatty alcohols, resulting in long-chain ester waxes ranging from C_38_ to C_56_, with a strong predominance of the even-atom carbon number homologues. The identification of the different esters was based on their mass spectra.^4,45^ The mass spectra of these compounds typically display one or more intense peaks corresponding to protonated acid ions, offering valuable insight into the acid moieties. The alcohol moieties are identified through the molecular ion peak, which reveals the total number of carbon atoms in the ester. By combining the information on the ester’s total carbon count with the data on the acid moiety, the identity of the alcohol moiety can be readily deduced by subtraction. Quantification of each ester was achieved by integrating the chromatographic peak areas of the characteristic ions for each acid moiety. The detailed composition of the high molecular-weight esters identified in the cereal straws is shown in Table 2. The esterified *n*-fatty acids ranged from tetradecanoic acid (C_14_) to octacosanoic acid (C_28_), while the esterified *n*-fatty alcohols ranged from eicosanol (C_20_) to dotriacontanol (C_32_). Despite the high abundance of free linoleic and oleic acids in the straw samples, the absence of unsaturated *n*-fatty acid forming high molecular-weight esters was particularly striking. The most abundant high molecular-weight ester was C_44_, mainly composed of hexadecanoic acid, octacosyl ester (C_16_:C_28_) (**XVII**) in triticale (639 mg/kg), wheat (241 mg/kg), and tritordeum (104 mg/kg). In rye, however, the most abundant high molecular-weight ester was C_42_, mainly composed of hexadecanoic acid, hexacosyl ester (C_16_:C_26_), accounting for 127 mg/kg (Table 1). A similar pattern was observed in a previous work on wheat straw.^4^ Waxes are essential components in the plant cuticle, acting as a protective barrier between the plant surface and the external environment. They protect the plant from pathogens, water loss, and ultraviolet radiation. Due to these properties, waxes have a wide range of industrial applications, including in pharmacology as well as lubricants.^46,47^

**Table 2:** Composition and Abundance (mg/kg) of the High Molecular-Weight Esters Identified in the Acetone Extracts from Wheat, Triticale, Rye, and Tritordeum Straws

**compound**	**fatty acid:fatty alcohol**	**wheat**	**triticale**	**rye**	**tritordeum**
**esters C**_**38**_		21 ± 3	23 ± 1	5 ± 0	17 ± 1
tetradecanoic acid, tetracosyl ester	C14:C24	9 ± 1	7 ± 0	2 ± 0	6 ± 0
hexadecanoic acid, docosyl ester	C16:C22	12 ± 2	16 ± 1	3 ± 0	11 ± 1
**esters C**_**39**_		5 ± 1	3 ± 0	1 ± 0	3 ± 0
pentadecanoic acid, tetracosyl ester	C15:C24	3 ± 1	1 ± 0	tr.	n.d.
hexadecanoic acid, tricosyl ester	C16:C23	3 ± 0	2 ± 0	1 ± 0	n.d.
heptadecanoic acid, docosyl ester	C17:C22	n.d.	n.d.	n.d.	3 ± 0
octadecanoic acid, heneicosyl ester	C18:C21	tr.	n.d.	n.d.	n.d.
**esters C**_**40**_		128 ± 4	89 ± 5	48 ± 2	50 ± 3
tetradecanoic acid, hexacosyl ester	C14:C26	7 ± 2	12 ± 0	23 ± 1	8 ± 1
hexadecanoic acid, tetracosyl ester	C16:C24	115 ± 1	72 ± 5	25 ± 1	38 ± 1
octadecanoic acid, docosyl ester	C18:C22	4 ± 1	5 ± 0	n.d.	3 ± 1
eicosanoic acid, eicosyl ester	C20:C20	2 ± 0	n.d.	n.d.	2 ± 0
**esters C**_**41**_		11 ± 1	9 ± 1	4 ± 1	7 ± 1
pentadecanoic acid, hexacosyl ester	C15:C26	2 ± 0	4 ± 1	3 ± 1	n.d.
hexadecanoic acid, pentacosyl ester	C16:C25	7 ± 1	5 ± 0	1 ± 0	7 ± 1
heptadecanoic acid, tetracosyl ester	C17:C24	2 ± 0	n.d.	n.d.	n.d.
**esters C**_**42**_		120 ± 4	218 ± 14	149 ± 17	94 ± 2
tetradecanoic acid, octacosyl ester	C14:C28	43 ± 2	95 ± 7	5 ± 0	24 ± 1
hexadecanoic acid, hexacosyl ester	C16:C26	46 ± 1	94 ± 6	127 ± 13	44 ± 1
octadecanoic acid, tetracosyl ester	C18:C24	17 ± 0	12 ± 0	9 ± 2	8 ± 0
eicosanoic acid, docosyl ester	C20:C22	14 ± 1	16 ± 0	8 ± 2	15 ± 0
docosanoic acid, eicosyl ester	C22:C20	n.d.	1 ± 0	n.d.	3 ± 0
**esters C**_**43**_		19 ± 1	32 ± 4	5 ± 1	9 ± 1
pentadecanoic acid, octacosyl ester	C15:C28	14 ± 1	22 ± 2	n.d.	4 ± 0
hexadecanoic acid, heptacosyl ester	C16:C27	5 ± 0	10 ± 2	n.d.	5 ± 1
heptadecanoic acid, hexacosyl ester	C17:C26	n.d.	n.d.	5 ± 1	n.d.
**esters C**_**44**_		311 ± 3	699 ± 32	81 ± 8	158 ± 15
tetradecanoic acid, triacontyl ester	C14:C30	4 ± 0	7 ± 0	n.d.	3 ± 0
hexadecanoic acid, octacosyl ester (**XVII**)	C16:C28	241 ± 10	639 ± 26	27 ± 2	104 ± 12
octadecanoic acid, hexacosyl ester	C18:C26	6 ± 2	15 ± 2	23 ± 3	9 ± 1
eicosanoic acid, tetracosyl ester	C20:C24	37 ± 4	23 ± 2	26 ± 3	28 ± 1
docosanoic acid, docosyl ester	C22:C22	15 ± 5	15 ± 2	50 ± 0	12 ± 1
tetracosanoic acid, eicosyl ester	C24:C20	n.d.	n.d.	n.d.	3 ± 0
**esters C**_**45**_		15 ± 1	16 ± 0	5 ± 1	13 ± 1
hexadecanoic acid, nonacosyl ester	C16:C29	12 ± 1	7 ± 0	4 ± 1	5 ± 0
heptadecanoic acid, octacosyl ester	C17:C28	3 ± 0	9 ± 0	1 ± 0	8 ± 1
**esters C**_**46**_		108 ± 10	104 ± 9	64 ± 7	65 ± 4
hexadecanoic acid, triacontyl ester	C16:C30	27 ± 2	30 ± 3	n.d.	11 ± 1
octadecanoic acid, octacosyl ester	C18:C28	34 ± 2	36 ± 3	5 ± 1	12 ± 1
eicosanoic acid, hexacosyl ester	C20:C26	10 ± 0	19 ± 0	45 ± 4	11 ± 1
docosanoic acid, tetracosyl ester	C22:C24	37 ± 6	13 ± 2	14 ± 2	24 ± 1
tetracosanoic acid, docosyl ester	C24:C22	n.d.	8 ± 1	n.d.	7 ± 0
**esters C**_**48**_		75 ± 4	142 ± 13	42 ± 2	58 ± 1
hexadecanoic acid, dotriacontyl ester	C16:C32	n.d.	11 ± 2	n.d.	8 ± 0
octadecanoic acid, triacontyl ester	C18:C30	n.d.	4 ± 1	n.d.	n.d.
eicosanoic acid, octacosyl ester	C20:C28	49 ± 2	101 ± 7	8 ± 0	26 ± 1
docosanoic acid, hexacosyl ester	C22:C26	n.d.	15 ± 0	18 ± 2	15 ± 0
tetracosanoic acid, tetracosyl ester	C24:C24	10 ± 0	13 ± 3	17 ± 0	11 ± 0
hexacosanoic acid, docosyl ester	C26:C22	16 ± 2	n.d.	n.d.	n.d. ±
**ester C**_**50**_		71 ± 5	117 ± 11	24 ± 1	49 ± 2
docosanoic acid, octacosyl ester	C22:C28	54 ± 3	101 ± 10	5 ± 0	33 ± 2
tetracosanoic acid, hexacosyl ester	C24:C26	17 ± 2	7 ± 0	13 ± 1	8 ± 0
hexacosanoic acid, tetracosyl ester	C26:C24	n.d.	9 ± 1	5 ± 0	8 ± 0
**esters C**_**52**_		35 ± 3	50 ± 6	16 ± 1	26 ± 1
tetracosanoic acid, octacosyl ester	C24:C28	29 ± 2	46 ± 6	2 ± 0	15 ± 1
hexacosanoic acid, hexacosyl ester	C26:C26	n.d.	4 ± 0	14 ± 1	5 ± 0
octacosanoic acid, tetracosyl ester	C28:C24	16 ± 1	n.d.	n.d.	6 ± 0
**ester C**_**54**_		16 ± 1	26 ± 1	**n.d.**	13 ± 0
hexacosanoic acid, octacosyl ester	C26:C28	16 ± 1	26 ± 1	n.d.	13 ± 0
**esters C**_**56**_		24 ± 1	32 ± 3	**n.d.**	10 ± 3
octacosanoic acid, octacosyl ester	C28:C28	24 ± 1	32 ± 3	n.d.	10 ± 3

### 3.3 Steroid Compounds

The steroidal compounds identified in the selected cereal straws can be categorized into the following families: free sterols, sterol esters, sterol glycosides, steroid ketones, and steroid hydrocarbons. These compounds were present at varying concentrations, with the highest levels found in triticale (1954 mg/kg), followed by tritordeum (1746 mg/kg), wheat (1515 mg/kg), and rye (1358 mg/kg) (Table 1).

Free sterols were one of the major steroid families found in the straws, constituting up to 1192 mg/kg (9.6% of all lipophilic compounds) in triticale, 1010 mg/kg (15.6%) in tritordeum, 860 mg/kg (13.1%) in wheat, and 824 mg/kg (15.5%) in rye (Figure 5). The most abundant free sterol was sitosterol (**XVIII**), with concentrations of 684 mg/kg in triticale, 563 mg/kg in tritordeum, 496 mg/kg in rye, and 417 mg/kg in wheat (Table 1). Campesterol (**XIX**) and stigmasterol (**XX**) were also found in significant amounts, with campesterol being more abundant, reaching up to 224 mg/kg in triticale, 202 mg/kg in rye, 196 mg/kg in wheat, and 189 mg/kg in tritordeum. Stigmasterol was less abundant, accounting for 161 mg/kg in wheat, 147 mg/kg in tritordeum, 105 mg/kg in triticale, and 48 mg/kg in rye (Table 1). Most of the sterols identified were common across all cereal straws. However, certain sterols, such as Δ^5^-avenasterol (**XXI**), cycloartenol (**XXII**), and 24-methylenecycloartanol (**XXIII**), were absent in rye, whereas Δ^7^-stigmastenol (**XXIV**) was detected exclusively in rye. These differences likely arise from the close genetic relationship among wheat, triticale, and tritordeum. These findings broaden the range of sterols identified in cereal straws, which were previously limited to campesterol, stigmasterol, and sitosterol in the literature.^4,19^

Sterol esters were identified in lower amounts, accounting for 122 mg/kg (0.9% of all lipophilic compounds) in triticale, 73 mg/kg (1.1%) in wheat, 47 mg/kg (0.8%) in tritordeum, and 35 mg/kg (0.7%) in rye (Figure 5). Sterol esters were formed from three sterols—campesterol, stigmasterol, and sitosterol—esterified to various long-chain fatty acids. The identities of the sterol esters were determined through the prominent base peaks in their mass spectra, which corresponded to the steroid components (*m*/*z* 382 for campesterol, *m*/*z* 394 for stigmasterol, and *m*/*z* 396 for sitosterol). Each of these sterols was esterified with fatty acids ranging from tetradecanoic acid (C_14_) to octadecanoic acid (C_18_), including unsaturated oleic (C_18:1_) and linoleic (C_18:2_) acids. Sitosterol esters were the most prevalent, with sitosteryl tetradecanoate (C_14_; **XXV**) being the most abundant, with concentrations of 33 mg/kg in wheat, 32 mg/kg in triticale, 25 mg/kg in tritordeum, and 18 mg/kg in rye (Table 1).

**Figure 7:**
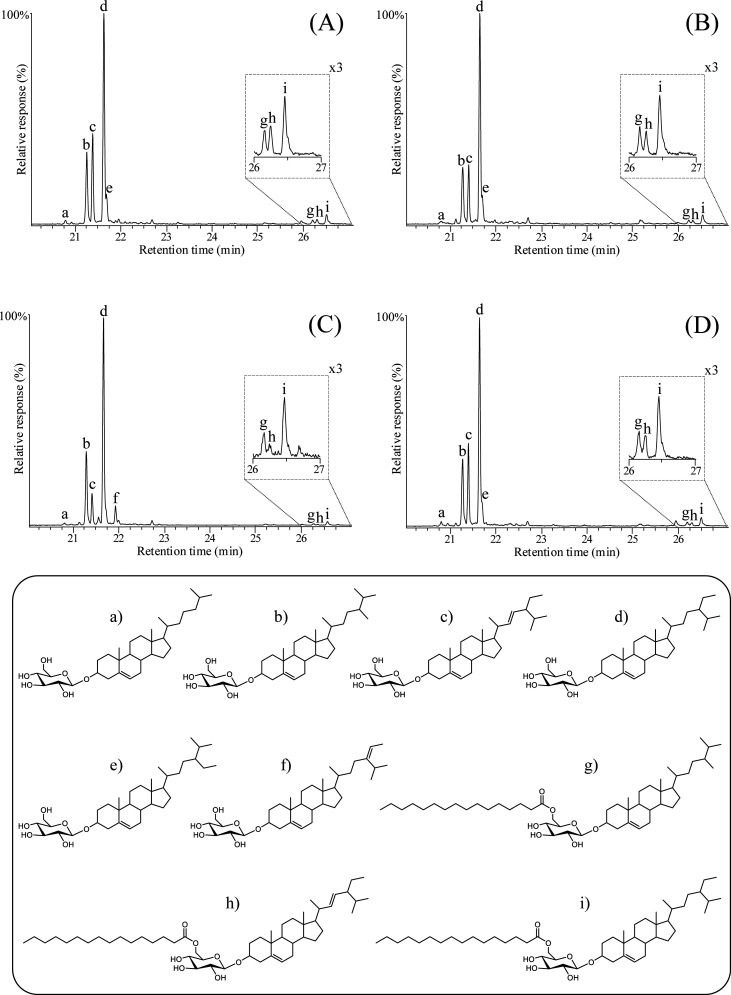
Single-ion (*m*/*z* 204) chromatograms of wheat (A), triticale (B), rye (C), and tritordeum (D), showing the distribution of the different sterol glycosides and acyl sterol glycosides. The identified compounds, and their corresponding structures shown at the bottom, are labeled as follows: (a) cholesteryl 3β-d-glucopyranoside, (b) campesteryl 3β-d-glucopyranoside, (c) stigmasteryl 3β-d-glucopyranoside, (d) sitosteryl 3β-d-glucopyranoside (**XXVI**), (e) Δ^7^-stigmastenyl 3β-d-glucopyranoside, (f) Δ^5^-avenasteryl 3β-d-glucopyranoside, (g) campesteryl (6′-*O*-palmitoyl) 3β-d-glucopyranoside, (h) stigmasteryl (6′-*O*-palmitoyl) 3β-d-glucopyranoside, (i) sitosteryl (6′-*O*-palmitoyl) 3β-d-glucopyranoside (**XXVII**).

Sterol glycosides and acyl sterol glycosides made up the second most abundant family of steroid compounds found in the analyzed cereal straws. They accounted for 649 mg/kg (10.0% of the total lipophilic compounds) in tritordeum, 599 mg/kg (4.9%) in triticale, 550 mg/kg (8.4%) in wheat, and 481 mg/kg (9.0%) in rye, as shown in Figure 5. Sitosteryl 3β-d-glucopyranoside (**XXVI**) was the most abundant sterol glycoside, accounting for 335 mg/kg in tritordeum, 328 mg/kg in triticale, 279 mg/kg in rye, and 273 mg/kg in wheat (Table 1). Campesteryl and stigmasteryl 3β-d-glucopyranosides were also detected in considerable amounts, accounting for 129 and 114 mg/kg in tritordeum, 94 and 115 mg/kg in wheat, 108 and 89 mg/kg in triticale, and 107 and 58 mg/kg in rye (Table 1). Similar to free sterols, Δ^5^-avenasteryl 3β-d-glucopyranoside was identified in wheat (15 mg/kg), tritordeum (9 mg/kg), and triticale (7 mg/kg), while Δ^7^-stigmastenyl 3β-d-glucopyranoside was identified only in rye (10 mg/kg). Various acyl sterol glycosides were identified in cereal straws. The identification of these compounds was carried out by comparing the mass spectra and retention time with authentic standards (as their TMS-ether derivatives), as previously described.^48^ The most abundant one was sitosteryl (6′-*O*-palmitoyl)-3β-d-glucopyranoside (**XXVII**), ranging from 32 mg/kg in triticale to 15 mg/kg in rye straw. Campesteryl and stigmasteryl (6′-*O*-palmitoyl)-3β-d-glucopyranosides were also detected, although in much lower amounts (Table 1). Figure 7 presents relevant sections of the single-ion *m*/*z* 204 (the base peak for sterol glycosides and acyl sterol glycosides) chromatograms of the four cereal straws, illustrating the distribution of these compounds in the chloroform extracts of the straws. Again, wheat, triticale, and tritordeum followed a similar pattern due to their close relationship. Sterol glycosides are highly valuable for their ability to reduce cholesterol absorption in humans.^49^ As with free sterols, the current findings expand the range of these compounds in cereal straws beyond those reported in previous work, namely, campesteryl, stigmasteryl, and sitosteryl 3β-d-glucopyranosides.^4^

Finally, minor amounts of steroidal ketones and steroid hydrocarbons were detected. Steroid ketones accounted for 26 mg/kg in tritordeum, 22 mg/kg in triticale, 20 mg/kg in wheat, and 7 mg/kg in rye (Table 1). The steroid ketones identified were stigmastane-3,6-dione (**XXVIII**), ergost-4-en-3-one (**XXIX**), stigmasta-3,5-dien-7-one (**XXX**), and stigmast-4-en-3-one (**XXXI**), which had been reported in previous studies.^4^ Steroid hydrocarbons were found ranging from 19 mg/kg in triticale, 14 mg/kg in tritordeum, 12 mg/kg in wheat, and 11 mg/kg in rye (Table 1). Only two compounds were identified, stigmasta-3,5,22-triene (**XXXII**), which was present in higher amounts (6–11 mg/kg), and stigmasta-3,5-diene (**XXXIII**), which was found in lower amounts (4–8 mg/kg). These compounds are likely degradation products of free and conjugated sterols, as previously described.^50^

In conclusion, the lipophilic extracts from straws of four cereal species (wheat, triticale, rye, and tritordeum) were comprehensively analyzed. The predominant compounds identified included *n*-fatty acids, β-diketones, steroid compounds, high molecular-weight esters, and *n*-fatty alcohols. Series of *n*-alkanes, phytol and phytyl esters, 2-hydroxyfatty acids, acylglycerides, tocopherols and tocopheryl esters, and *n*-aldehydes were also detected, albeit in lower amounts. These compound families are highly promising in the nutraceutical, chemical, and pharmaceutical industries, presenting opportunities to extract valuable phytochemicals of significant value from agricultural residues. Triticale straw was distinguished by its exceptionally high levels of key lipophilic compounds, including *n*-fatty acids, β-diketones, *n*-fatty alcohols, free sterols, and high molecular-weight esters. These values align with its notably high total lipophilic content, underscoring its potential for diverse applications in biobased industries. Particularly noteworthy was the β-diketone hentriacontane-14,16-dione, which emerged as the most abundant compound in the four straw samples analyzed. This compound holds significant potential for numerous applications in various industries, making cereal straw an optimal source for its extraction. Beyond potential variations influenced by growing conditions and the environment, the findings of this study offer valuable information to enhance the value of these abundant and low-cost agricultural residues, positioning them as versatile feedstock in lignocellulosic biorefineries.

## References

[ref1] Gani A.; Sm W.; Fa M. Whole-Grain Cereal Bioactive Compounds and Their Health Benefits: A Review. J. Food Process. Technol. 2012, 3 (3), 1000146 10.4172/2157-7110.1000146.

[ref2] FAOSTAT. Food and Agriculture Organization of the United Nations (FAO). https://www.fao.org/faostat.

[ref3] Lafiandra D.; Riccardi G.; Shewry P. R. Improving Cereal Grain Carbohydrates for Diet and Health. J. Cereal Sci. 2014, 59 (3), 312–326. 10.1016/j.jcs.2014.01.001. 24966450 PMC4064937

[ref4] del Río J. C.; Prinsen P.; Gutiérrez A. A Comprehensive Characterization of Lipids in Wheat Straw. J. Agric. Food Chem. 2013, 61 (8), 1904–1913. 10.1021/jf304252m. 23373527

[ref5] Marques G.; Gutiérrez A.; Barro F.; del Río J. C.; Rencoret J. Seasonal Variability of Lipophilic Compounds in Oat (*Avena sativa* L.) Straw: A Comprehensive Chemical Study. J. Agric. Food Chem. 2024, 72 (36), 19891–19903. 10.1021/acs.jafc.4c05002. 39225266 PMC11403623

[ref6] Xu F. Chapter 2 - Structure, Ultrastructure, and Chemical Composition. In Cereal Straw as a Resource for Sustainable Biomaterials and Biofuels; Sun R.-C., Ed.; Elsevier: Amsterdam, 2010; pp 9–47. 10.1016/B978-0-444-53234-3.00002-X.

[ref7] Ghosh S.; Chowdhury R.; Bhattacharya P. Sustainability of Cereal Straws for the Fermentative Production of Second Generation Biofuels: A Review of the Efficiency and Economics of Biochemical Pretreatment Processes. Appl. Energy 2017, 198, 284–298. 10.1016/j.apenergy.2016.12.091.

[ref8] Karp S. G.; Sydney E. B.; Woiciechowski A. L.; Letti L. A. J.; De Carvalho J. C.; Torres L. A. Z.; Kumlehn G. S.; De Souza Candeo E.; Soccol C. R. Lignocellulosic Biorefinery for Value-Added Products: The Emerging Bioeconomy. In Biomass, Biofuels, Biochemicals; Elsevier, 2021; pp 291–321. 10.1016/B978-0-12-821878-5.00002-7.

[ref9] Rosado M. J.; Marques G.; Rencoret J.; Gutiérrez A.; del Río J. C. Chemical Composition of Lipophilic Compounds From rice (*Oryza sativa*) Straw: An Attractive Feedstock for Obtaining Valuable Phytochemicals. Front. Plant Sci. 2022, 13, 868319 10.3389/fpls.2022.868319. 35392522 PMC8981202

[ref10] Bajwa D. S.; Pourhashem G.; Ullah A. H.; Bajwa S. G. A Concise Review of Current Lignin Production, Applications, Products and Their Environmental Impact. Ind. Crops Prod. 2019, 139, 111526 10.1016/j.indcrop.2019.111526.

[ref11] Mujtaba M.; Fernandes Fraceto L.; Fazeli M.; Mukherjee S.; Savassa S. M.; Araujo De Medeiros G.; Do Espírito Santo Pereira A.; Mancini S. D.; Lipponen J.; Vilaplana F. Lignocellulosic Biomass from Agricultural Waste to the Circular Economy: A Review with Focus on Biofuels, Biocomposites and Bioplastics. J. Clean. Prod. 2023, 402, 136815 10.1016/j.jclepro.2023.136815.

[ref12] Singh N.; Singhania R. R.; Nigam P. S.; Dong C.-D.; Patel A. K.; Puri M. Global Status of Lignocellulosic Biorefinery: Challenges and Perspectives. Bioresour. Technol. 2022, 344, 126415 10.1016/j.biortech.2021.126415. 34838977

[ref13] Verkasalo E.; Möttönen V.; Roitto M.; Vepsäläinen J.; Kumar A.; Ilvesniemi H.; Siwale W.; Julkunen-Tiitto R.; Raatikainen O.; Sikanen L. Extractives of Stemwood and Sawmill Residues of Scots Pine (*Pinus sylvestris* L.) for Biorefining in Four Climatic Regions in Finland—Phenolic and Resin Acid Compounds. Forests 2021, 12 (2), 192 10.3390/f12020192.

[ref14] Kritchevsky D.; Chen S. C. Phytosterols—Health Benefits and Potential Concerns: A Review. Nutr. Res. (N.Y.) 2005, 25 (5), 413–428. 10.1016/j.nutres.2005.02.003.

[ref15] Ogbe R. J.; Ochalefu D. O.; Mafulul S. G.; Olaniru O. B. A Review on Dietary Phytosterols: Their Occurrence, Metabolism and Health Benefits. Asian J. Plant Sci. 2015, 5, 10.

[ref16] Traber M. G.; Atkinson J. Vitamin E, Antioxidant and Nothing More. Free Radic. Biol. Med. 2007, 43 (1), 4–15. 10.1016/j.freeradbiomed.2007.03.024. 17561088 PMC2040110

[ref17] Tidiane Sall A.; Chiari T.; Legesse W.; Seid-Ahmed K.; Ortiz R.; Van Ginkel M.; Bassi F. M. Durum Wheat (*Triticum durum* Desf.): Origin, Cultivation and Potential Expansion in Sub-Saharan Africa. Agronomy 2019, 9 (5), 263 10.3390/agronomy9050263.

[ref18] Mergoum M.; Singh P. K.; Peña R. J.; Lozano-del Río A. J.; Cooper K. V.; Salmon D. F.; Macpherson H. g. Triticale: A “New” Crop with Old Challenges. In Cereals; Carena M. J., Ed.; Springer US: New York, NY, 2009; pp 267–287. 10.1007/978-0-387-72297-9_9.

[ref19] Athukorala Y.; Mazza G. Supercritical Carbon Dioxide and Hexane Extraction of Wax from Triticale Straw: Content, Composition and Thermal Properties. Ind. Crops Prod. 2010, 31 (3), 550–556. 10.1016/j.indcrop.2010.02.011.

[ref20] Drzymała K.; Mirończuk A. M.; Pietrzak W.; Dobrowolski A. Rye and Oat Agricultural Wastes as Substrate Candidates for Biomass Production of the Non-Conventional Yeast *Yarrowia Lipolytica*. Sustainability 2020, 12 (18), 7704 10.3390/su12187704.

[ref21] Wiesenberg G. L. B.; Lehndorff E.; Schwark L. Thermal Degradation of Rye and Maize Straw: Lipid Pattern Changes as a Function of Temperature. Org. Geochem. 2009, 40 (2), 167–174. 10.1016/j.orggeochem.2008.11.004.

[ref22] del Río J. C.; Evaristo A. B.; Marques G.; Martín-Ramos P.; Martín-Gil J.; Gutiérrez A. Chemical Composition and Thermal Behavior of the Pulp and Kernel Oils from Macauba Palm (*Acrocomia aculeata*) Fruit. Ind. Crops Prod. 2016, 84, 294–304. 10.1016/j.indcrop.2016.02.018.

[ref23] Gutiérrez A.; del Río J. C.; González-Vila F. J.; Martín F. Analysis of Lipophilic Extractives from Wood and Pitch Deposits by Solid-Phase Extraction and Gas Chromatography. J. Chromatogr. A 1998, 823 (1–2), 449–455. 10.1016/S0021-9673(98)00356-2.

[ref24] Gutiérrez A.; del Río J. C.; Martínez Á. T. Chemical Analysis and Biological Removal of Wood Lipids Forming Pitch Deposits in Paper Pulp Manufacturing. In Environmental Microbiology: Methods and Protocols; Walker J. M., Spencer J. F. T., Ragout de Spencer A. L., Eds.; Humana Press: Totowa, NJ, 2004; pp 189–202. 10.1385/1-59259-765-3:189.

[ref25] Rosado M. J.; Marques G.; Rencoret J.; Gutiérrez A.; Bausch F.; Rosenau T.; Potthast A.; del Río J. C. Chemical Composition of the Lipophilic Compounds from the Rind and Pith of Papyrus (*Cyperus papyrus* L.) Stems. Front. Plant Sci. 2022, 13, 1097866 10.3389/fpls.2022.1097866. 36618622 PMC9813494

[ref26] Marques G.; Gutiérrez A.; del Río J. C. Chemical Characterization of Lignin and Lipophilic Fractions from Leaf Fibers of Curaua (*Ananas erectifolius*). J. Agric. Food Chem. 2007, 55 (4), 1327–1336. 10.1021/jf062677x. 17253715

[ref27] Carballeira N. M.; Shalabi F.; Reyes M. New 2-Hydroxy Fatty Acids in the Caribbean Urchin Tripneustes Esculentus. J. Nat. Prod. 1994, 57 (5), 614–619. 10.1021/np50107a008. 8064293

[ref28] Indira M.; Peele K. A.; Krupanidhi S.; Prabhakar K. V.; Vimala K. B. S.; Kavya P. S.; Sravya I.; Venkateswarulu T. C. In Vitro Assessment of the Bioactive Compounds and Anticancer Potential of *Citrus medica* Leaf Extract. Trop. Life Sci. Res. 2023, 34 (3), 197 10.21315/tlsr2023.34.3.11. PMC10583853 37860090

[ref29] Islam M. T.; Ali E. S.; Uddin S. J.; Shaw S.; Islam M. A.; Ahmed M. I.; Chandra Shill M.; Karmakar U. K.; Yarla N. S.; Khan I. N.; Billah M. M.; Pieczynska M. D.; Zengin G.; Malainer C.; Nicoletti F.; Gulei D.; Berindan-Neagoe I.; Apostolov A.; Banach M.; Yeung A. W. K.; El-Demerdash A.; Xiao J.; Dey P.; Yele S.; Jóźwik A.; Strzałkowska N.; Marchewka J.; Rengasamy K. R. R.; Horbańczuk J.; Kamal M. A.; Mubarak M. S.; Mishra S. K.; Shilpi J. A.; Atanasov A. G. Phytol: A Review of Biomedical Activities. Food Chem. Toxicol. 2018, 121, 82–94. 10.1016/j.fct.2018.08.032. 30130593

[ref30] Olofsson P.; Hultqvist M.; Hellgren L. I.; Holmdahl R. Phytol: A Chlorophyll Component with Anti-Inflammatory and Metabolic Properties. In Recent Advances in Redox Active Plant and Microbial Products: From Basic Chemistry to Widespread Applications in Medicine and Agriculture; Jacob C., Kirsch G., Slusarenko A., Winyard P. G., Burkholz T., Eds.; Springer Netherlands: Dordrecht, 2014; pp 345–359. 10.1007/978-94-017-8953-0_13.

[ref31] Asemave K.; Byrne F. P.; Clark J. H.; Farmer T. J.; Hunt A. J. Modification of Bio-Based β-Diketone from Wheat Straw Wax: Synthesis of Polydentate Lipophilic Super-Chelators for Enhanced Metal Recovery. RSC Adv. 2019, 9 (7), 3542–3549. 10.1039/C8RA09426H. 35518071 PMC9060256

[ref32] Noppawan P.; Sangon S.; Chatsiri P.; Khunmood N.; Aintharabunya S.; Supanchaiyamat N.; Hunt A. J. Sustainable Solvents for β-Diketone Extraction from Wheat Straw Wax and Their Molecular Self-Assembly into Nano-Structured Tubules for Hydrophobic Coatings. RSC Adv. 2023, 13 (4), 2427–2437. 10.1039/D2RA07581D. 36741189 PMC9844676

[ref33] Tulloch A. P. Epicuticular Waxes from *Agropyron dasystachyum*, *Agropyron riparium* and *Agropyron elongatum*. Phytochemistry 1983, 22 (7), 1605–1613. 10.1016/0031-9422(83)80097-1.

[ref34] Meusel I.; Neinhuis C.; Markstädter C.; Barthlott W. Chemical Composition and Recrystallization of Epicuticular Waxes: Coiled Rodlets and Tubules. Plant Biol. 2000, 2 (4), 462–470. 10.1055/s-2000-5961.

[ref35] Kotha R. R.; Luthria D. L. Curcumin: Biological, Pharmaceutical, Nutraceutical, and Analytical Aspects. Molecules 2019, 24 (16), 2930 10.3390/molecules24162930. 31412624 PMC6720683

[ref36] Rahmani A.; Alsahli M.; Aly S.; Khan M.; Aldebasi Y. Role of Curcumin in Disease Prevention and Treatment. Adv. Biomed. Res. 2018, 7 (1), 38 10.4103/abr.abr_147_16. 29629341 PMC5852989

[ref37] Urbaniak W.; Jurek K.; Witt K.; Gorączko A. Properties and Application of Diketones and Their Derivatives. Chemik 2011, 65 (4), 273–282.

[ref38] Tulloch A. P.; Hoffman L. L. Epicuticular Waxes of Secale Cereale and Triticale Hexaploide Leaves. Phytochemistry 1974, 13 (11), 2535–2540. 10.1016/S0031-9422(00)86932-0.

[ref39] Gampe C.; Verma V. A. Curse or Cure? A Perspective on the Developability of Aldehydes as Active Pharmaceutical Ingredients. J. Med. Chem. 2020, 63 (23), 14357–14381. 10.1021/acs.jmedchem.0c01177. 32916044

[ref40] Bisignano G.; Laganà M. G.; Trombetta D.; Arena S.; Nostro A.; Uccella N.; Mazzanti G.; Saija A. In Vitro Antibacterial Activity of Some Aliphatic Aldehydes from *Olea Europaea* L. FEMS Microbiol. Lett. 2001, 198 (1), 9–13. 10.1111/j.1574-6968.2001.tb10611.x. 11325546

[ref41] Trombetta D.; Saija A.; Bisignano G.; Arena S.; Caruso S.; Mazzanti G.; Uccella N.; Castelli F. Study on the Mechanisms of the Antibacterial Action of Some Plant α,β-Unsaturated Aldehydes. Lett. Appl. Microbiol. 2002, 35 (4), 285–290. 10.1046/j.1472-765X.2002.01190.x. 12358689

[ref42] del Río J. C.; Marques G.; Lino A. G.; Lima C. F.; Colodette J. L.; Gutiérrez A. Lipophilic Phytochemicals from Sugarcane Bagasse and Straw. Ind. Crops Prod. 2015, 77, 992–1000. 10.1016/j.indcrop.2015.09.064.

[ref43] Hussain N.; Ishak I.; Coorey R.; Ghani M. A.; Ping T. C. Chapter 31-Tocopherols. In A Centum of Valuable Plant Bioactives; Elsevier, 2021; pp 707–731. 10.1016/B978-0-12-822923-1.00011-X.

[ref44] Shahidi F.; Ambigaipalan P. Omega-3 Polyunsaturated Fatty Acids and Their Health Benefits. Annu. Rev. Food Sci. Technol. 2018, 9 (1), 345–381. 10.1146/annurev-food-111317-095850. 29350557

[ref45] del Río J. C.; Marques G.; Rodríguez I. M.; Gutiérrez A. Chemical Composition of Lipophilic Extractives from Jute (*Corchorus capsularis*) Fibers Used for Manufacturing of High-Quality Paper Pulps. Ind. Crops Prod. 2009, 30 (2), 241–249. 10.1016/j.indcrop.2009.04.001.

[ref46] Shirani A.; Joy T.; Lager I.; Yilmaz J. L.; Wang H.-L.; Jeppson S.; Cahoon E. B.; Chapman K.; Stymne S.; Berman D. Lubrication Characteristics of Wax Esters from Oils Produced by a Genetically-Enhanced Oilseed Crop. Tribol. Int. 2020, 146, 106234 10.1016/j.triboint.2020.106234.

[ref47] Zeisler-Diehl V. V.; Barthlott W.; Schreiber L. Plant Cuticular Waxes: Composition, Function, and Interactions with Microorganisms. In Hydrocarbons, Oils and Lipids: Diversity, Origin, Chemistry and Fate; Wilkes H., Ed.; Springer International Publishing: Cham, 2020; pp 123–138. 10.1007/978-3-319-90569-3_7.

[ref48] Gutiérrez A.; del Río J. C. Gas Chromatography/Mass Spectrometry Demonstration of Steryl Glycosides in Eucalypt Wood, Kraft Pulp and Process Liquids. Rapid Commun. Mass Spectrom. 2001, 15 (24), 2515–2520. 10.1002/rcm.537. 11746925

[ref49] Lin X.; Ma L.; Racette S. B.; Anderson Spearie C. L.; Ostlund R. E. Phytosterol Glycosides Reduce Cholesterol Absorption in Humans. Am. J. Physiol.-Gastrointest. Liver Physiol. 2009, 296 (4), G931–G935. 10.1152/ajpgi.00001.2009. 19246636 PMC2670661

[ref50] Marques G.; Rencoret J.; Gutiérrez A.; del Río J. C. Lipophilic Compounds from Maize Fiber and Rice Husk Residues – An Abundant and Inexpensive Source of Valuable Phytochemicals. Ind. Crops Prod. 2020, 146, 112203 10.1016/j.indcrop.2020.112203.

